# Quantifying the “Slosh Stomach”: A Novel Tool for Assessment of Exercise-Associated Gastroparesis Symptoms in Endurance Athletes

**DOI:** 10.1155/2016/1276369

**Published:** 2016-11-17

**Authors:** Amy Sue Biondich, Jeremy D. Joslin

**Affiliations:** Department of Emergency Medicine, SUNY Upstate Medical University, Syracuse, NY, USA

## Abstract

*Introduction*. We describe a novel scale and its field use for evaluation of exercise-associated gastroparesis in the endurance athlete.* Methods*. A scale was created based on gastroparesis tools previously described in the medical literature. Surveys of the tool were administered to runners participating in a 210 km multiday foot race in Sri Lanka.* Results*. Use of this novel scale was demonstrated to be useful in assessing gastroparesis severity scores of athletes and how these symptoms affected their race performance. Of the 27 race participants who completed the survey, 27 felt that the tool adequately captured their symptoms.* Conclusions*. This novel survey tool was able to assess the presence and severity of exercise-associated gastroparesis symptoms in endurance racers in a remote location. This tool may be helpful with further research of the identification and management of gastroparesis and other gastrointestinal upset in the endurance race environment.

## 1. Introduction

Interest and participation in endurance events and ultramarathons has become increasingly popular [1, 2]. Attempts have been made to understand what factors not only impact athletic performance in these events but also ultimately delineate the finishers from the nonfinishers [3, 4]. The bulk of physiologic research concentrates on marathons and shorter running events. However, there has been an increasing body of work aimed solely at understanding medical issues specific to the endurance athlete during race day [5, 6]. These ultramarathons frequently occur in remote geography and often subject athletes to harsh environmental conditions.

Although the importance of physical activity to one's overall health cannot be overstated, it is well-known that strenuous exertion has a multitude of possibly detrimental effects on gastrointestinal function [7–10]. Several authors report data from endurance events describing gastrointestinal (GI) distress as a common and pervasive problem even if only temporary [4, 11–16].

The physiology behind the GI distress experienced by endurance events participants is not well-understood and is likely multifactorial [9]. Proposed mechanisms include the mechanical stress on the GI tract as a result of the repetitive pounding action of running [17, 18], reduced splanchnic blood flow as a product of changes of blood distribution during strenuous activity [10, 19], electrolyte disturbances (especially hyponatremia) from excessive fluid intake [20], or general malaise from dehydration [21–25].

An etiology of gastrointestinal distress that is gaining interest in the endurance foot-race industry is exercise-associated gastroparesis (EAG) which is colloquially known as “slosh stomach.” Although gastroparesis is well reported in the gastroenterology literature, there is a paucity of published literature specific to endurance athletic events; and there is no objective tool to quantitatively measure the severity of these symptoms. There are several validated gastroparesis severity scales utilized by gastroenterologists [26, 27], which are not completely applicable to the endurance athlete. Utilizing previously well-validated scoring symptoms, a modified and novel scale to capture GI symptoms in endurance racers was created and subsequently tested in the field.

The purpose of this prospective study was to trial the field expediency of a quantitative measurement tool for the common ailment of exercise-associated gastroparesis, as well as the satisfaction of the athlete and physician with its use.

## 2. Methods

Institutional Review Board approval was obtained (Upstate Medical University Proposal #722316) to survey consented athletes at the Wild Elephant Trail, a 6-day, multistage, remote ultramarathon that registered 52 runners (63% male and average age 48.9 years) and held its inaugural race in March 2015. The race covers 210 km through rural and remote Sri Lanka in the area between Yapahuwa Temple and Sigiriya Rock. Environmental conditions are typically tropical with average temperatures between 24°C and 32°C. Geography includes rolling hills, jungle climbs, and wet stretches of relatively flat paddy fields.

A convenience sample of athletes was surveyed by the event physician. Surveys were administered either when the athlete approached the medical team requesting medical care of gastrointestinal symptoms, or when the physician performed routine athlete health assessments and discovered a gastrointestinal complaint. Survey answers were collected just prior to any treatment or physician recommendations and then at time of reassessment. All medical management was based on current best practices and standard of care and was not altered for the purposes of the study. Surveys were made anonymous after posttreatment assessment was completed.

The survey instrument was adapted from previously published tools which have been used to measure symptom severity of gastroparesis in the context of chronic illness [26, 27]. From these previously described tools, we selected 6 symptoms which we felt were germane to the endurance athlete based on the collective authors' experience with hundreds of athletes over several years. Symptoms included in our tool were nausea, vomiting, loss of appetite, loss of desire to drink, abdominal “sloshing,” and abdominal pain. Once in the field, the severity of these 6 symptoms of interest was recorded on a 0–5 (none to unbearable) Likert scale. The total scores of all 6 symptoms were summated to produce a gastroparesis severity score (GSS). Finally, our instrument consisted of the time of initial assessment and reassessment, along with treatment provided and the activity being performed by the athlete at the time of the complaint (rest, exertion, and meals).

At the same time, a separate question of our survey used a Visual Analog Scale (VAS) to assess the more generic question “how much are the symptoms affecting your race performance?” where one end was “no problems at all” and the other end was “I'm thinking of dropping out.” The single mark on the VAS was measured in millimeters and converted to a percent.

After management of the symptoms by rest, medication, or watchful waiting, reassessment was performed using the same Likert scale for the same 6 symptoms of interest as well as the same VAS question and scale. The change in GSS was used to measure improvement in symptoms. The change in VAS was used to measure the athlete's perceived performance impact from these symptoms. Two new questions were also asked at this time. To the athlete, it was “Did you feel like this score adequately captured all of your symptoms as well as their severity?” and to the physician administering the survey it was “Did you feel like this score adequately captured the runner's symptoms?”

Survey results were analyzed descriptively with SPSS (version 22; SPSS Inc., Chicago, IL).

## 3. Results

A total of 27 surveys were obtained which included both the pretreatment and posttreatment assessments for athletes with gastroparesis symptoms. Surveys were administered to athletes either during exertion on the race course (16/27; 59%), during an attempt to take a meal (1/27; 4%), or after stage completion during recovery activities (10/27; 37%). No statistical differences were seen associating symptoms experienced with type of activity.

Summation of the severity scores (0–5 Likert scale) of all six symptoms was used to calculate a global gastroparesis severity score (GSS) of 0–30 for each athlete. The median pretreatment GSS was 6 (range: 2–16) with 23 athletes reporting improvement of their GSS and 4 athletes reporting no change or worsening of their symptoms. The median posttreatment GSS was 2 (range: 0–9).  Table 1 describes the percent of athletes who experienced each symptom at initial evaluation (before treatment) and at follow-up (after treatment), stratified by symptom severity on the Likert scale (0–5).

Reassessments were done at the convenience of the athlete and without time constraints given. The median time between assessments was 1:05 hours (range 00:05 hours to 9:55 hours; SD 2:33 hours). No statistical differences were seen associating GSS with time elapsed between assessments.

Medical management of athletes included anticipatory guidance (7/27; 26%), prescribed rest (13/27; 48%), or medication (7/27; 26%). Anticipatory guidance included suggestions for the athlete to shed their heat load by cooling themselves by rest or shade. Medications used included metoclopramide (1/27) and ondansetron (6/27). Median GSS decrease was 1 for anticipatory guidance, 2 for prescribed rest, and 5 for medication (*p* = 0.446).

Athletes with a GSS improvement of at least 3 points (top 50th percentile) had a median improvement in their VAS of 25.9% whereas athletes with a GSS improvement less than 3 points (lower 50th percentile) had a median improvement in their VAS of 7.14%.

Finally, all (100% of responses) athletes indicated that the survey instrument adequately captured their symptoms and severity. Similarly, the physician administering the surveys felt that, throughout the athlete encounters, the symptoms were adequately captured (100% of responses).

## 4. Discussion

Although this analysis does not specifically test sensitivity or specificity of the symptom domains, it does demonstrate that this tool can be used in the field to effectively capture the most common symptoms that occur with exercise-associated gastroparesis. The fact that athlete improvement in GSS was predictive of improvement of their VAS score suggests that the symptoms we chose were important to the athletes' race performance. Further psychometric validation would be needed to ascertain the strengths of each component, but because all of the athletes felt that their symptoms were captured, it is plausible that future analysis of this novel tool would demonstrate statistical sensitivity. Specificity of the symptoms would require a larger number of participants and a larger number of symptoms captured.

The severity of runner's symptoms was not associated with the activity that they were involved in at the time of questioning. The timing at which the runner was reassessed also did not have an association with the symptom score. Although this was not the primary objective of this study, it was noted that nausea and loss of appetite were associated most with the runner's overall attitude in their ability to complete the race. This finding may help guide future studies, including those that have to do with the treatment of EAG.

During this event, no athletes developed intractable symptoms that would prompt the race physician to consider other, more sinister, causes of nausea or fatigue. No athletes received intravenous fluids or required medical disqualification or evacuation. Information on “Did not finish” (DNF) status of athletes was not included in the data collected during this study. Though not statistically significant, there is a suggestion that medication may be more effective than simply rest alone.

Although EAG is a well-recognized and described syndrome in endurance medicine [10, 23–25], there has yet to be any symptom assessment instruments developed or any formal or extensive discussion of treatment published. Recent work has added greatly to our understanding of GI distress in the setting of endurance events by studying the incidence, severity, and timing of GI symptoms in finishers and nonfinishers of a 161 km race [16]; however a tool to discretely measure these symptoms in a race setting has yet to be developed. Although a formal validation study is still needed, results of our study suggest that our novel, field-expedient survey tool can be used to measure and assess the presence and severity of exercise-associated gastroparesis symptoms. The gastrointestinal symptoms chosen for our survey are widely considered to be common amongst endurance athletes.

Our novel EAG scale was developed by adapting other publications that have demonstrated the usefulness and validity of GI symptom severity scores [26, 27]. The symptoms chosen for inclusion in the survey were a result of previous gastroparesis research as well as the authors' experience with athletes in the race environment. Previous GI symptoms scores have included both discrete Likert-type questions for specific symptoms and a continuous VAS component attempting to capture more of a global understanding of the effects of the studied symptoms [26]. Though we utilized both elements to trial our tool, we have opted to only use the Likert questions in future iterations due to the ease of use. See Table 2 for a proposed EAG scale that does not include the VAS or other questions that were included for purposes of understanding the perception of this tool and its field expediency.

Limitations of this study involve those inherent in obtaining a convenience study sample. This limitation is difficult to control for in the remote, ultramarathon setting where athletes do not typically wish to participate in any kind of extraneous activities that may slow their competitive intents. The race physician who was queried by the survey was also the treating physician, which may introduce some ascertainment bias. Finally, because no athletes had any serious illness we cannot assess the utility of this tool in more critically ill athletes; however, this was not our intent and should not be the proposed target of the use of this tool.

This novel EAG score was developed in an attempt to provide researchers and clinicians with a tool to quantify the severity of this illness and response to therapy. To our knowledge no clinical trials have been conducted assessing the several treatments available for EAG. Further study is needed to determine if this questionnaire is valid, but our development of a scoring system to quantify largely subjective symptoms in an austere environment is not without precedent. For example, the Lake Louise Criteria for Acute Mountain Sickness were developed by expert consensus [28] and then trialed in the field to validate [29–31]. Our hope is that this expert-derived scoring system could derive further validation from future field use. One such immediate use would be to quantify symptoms in order to assess the effectiveness of medication-based interventions during races.

## 5. Conclusions

A simple Likert scale of the severity of nausea and loss of appetite seems to provide a useful tool for measuring the severity of exercise-associated gastroparesis in endurance athletes. Further research efforts focusing on this clinical syndrome may benefit from a numeric scoring system as described in this novel exercise-associated gastroparesis severity scale.

## Figures and Tables

**Table 1:** Percentage of patients reporting severity scores for each symptom before and after treatment. Scores that indicate improvement after treatment are shown in bold font.

	None (0)		Mild (1)		Moderate (2)		Severe (3)		Very severe (4)		Unbearable (5)	
	Initial	Follow-up	Initial	Follow-up	Initial	Follow-up	Initial	Follow-up	Initial	Follow-up	Initial	Follow-up
Nausea	0	22	26	44	48	**33**	19	**0**	7	**0**	0	0
Vomiting	70	96	26	**4**	4	**0**	0	0	0	0	0	0
Loss of appetite	11	41	44	**37**	22	**19**	19	**4**	4	**0**	0	0
Loss of desire to drink	22	59	41	**30**	30	**11**	4	**0**	4	**0**	0	0
Abdominal sloshing	67	96	11	**0**	22	**4**	0	0	0	0	0	0
Abdominal pain	78	85	22	**15**	0	0	0	0	0	0	0	0

**Table 2:** Final exercise-associated gastroparesis severity score.

Symptom	None	Mild	Moderate	Severe	Very severe	Unbearable
Nausea	0	1	2	3	4	5
Vomiting	0	1	2	3	4	5
Loss of appetite	0	1	2	3	4	5
Loss of desire to drink	0	1	2	3	4	5
Abdominal sloshing	0	1	2	3	4	5
Abdominal pain	0	1	2	3	4	5

Gastroparesis severity score (GSS) = sum of all circled numbers.
